# Expert insights on managing harmful algal blooms

**DOI:** 10.3389/ffwsc.2024.1452344

**Published:** 2024-10-02

**Authors:** Sarah Goodrich, Katherine Nicole Canfield, Kate Mulvaney

**Affiliations:** 1 Department of Geography and GIS, University of Cincinnati, Cincinnati, OH, United States; 2 U.S. Environmental Protection Agency, Office of Research and Development, Office of Science Advisor, Policy, and Engagement, Washington, DC, United States; 3 U.S. Environmental Protection Agency, Atlantic Coastal Environmental Sciences Division, Narragansett, RI, United States

**Keywords:** harmful algal blooms, interviews, water management, decision makers, monitoring, nutrients

## Abstract

Freshwater harmful algal blooms (HABs) are a global environmental, economic, social, and public health hazard. While there is an increasing understanding of the ecological considerations of HABs, there is limited understanding of human dimensions and management needs. We conducted semi-structured key informant interviews with 28 water managers and researchers to better understand how they perceive current management and to identify future management priorities in regard to HABs. For this study, we interviewed 31 key informants from three regions of the United States (New England, Ohio, and the Mountain West). We solicited insights across aspects of HABs management, including prevention, forecasting, monitoring, response, and communication. Nutrient management was the main consideration for the prevention of HAB events. Key informants noted that forecasting has the potential to be a valuable tool in the future but is not yet accurate enough at a local scale for widespread use. Monitoring was found to be implemented in varying ways across and even within the states and regions, with a need for more funding and standardization. HAB event responses vary greatly from chemical and physical treatments that suppress toxins to no-swim advisories, all with a mix of strengths and weaknesses. Finally, an increase in and improvement of communication efforts was identified as critical for reducing public health risks. These findings provide perceptions of current management practices and future plans while including opportunities to improve current freshwater HAB management efforts.

## Introduction

Excess nutrients introduced to waterbodies from anthropogenic activities such as fertilizer application and septic system use, can and often do lead to eutrophication and harmful algal blooms (HABs) throughout the United States and globally (Reinl et al., 2023; Olson et al., 2023; Malone and Newton, 2020; Gobler, 2019; Heil and Muni-Morgan, 2021). Eutrophication and HABs are further exacerbated by climate-induced warming of waterbodies. Climate change may also result in the changing of an ecosystem’s physical geography, making a location potentially more susceptible to HABs or a HAB event harder to control (Paerl et al., 2016). HABs are associated with a range of public health issues such as skin irritation, respiratory issues, vomiting, diarrhea, motor issues, and more depending on the HABs species and amount of exposure (Center for Disease Control Prevention, 2018). Fang et al. (2022) found 66% of global lakes identified by Landsat images showed a trend of HAB event frequency increase, with the most intense increase in North America. This same study identified fertilizer use and temperature as the primary anthropogenic activities contributing to HABs. Nutrient management has been identified as critical for HABs management, whether through an individual or community effort as well as industrial or large scale (Cheung et al., 2013; Sonak et al., 2018). HABs are predicted to, and in some cases have already demonstrated, increases in toxin production, frequency within a body of water, and potential to harm the aquatic ecosystem (Griffith and Gobler, 2019; Wells et al., 2015). This trend in increased cell densities is paired with earlier and longer summer warming of surface waters and increased total nitrogen concentrations (Smucker et al., 2021). In addition to threatening the aquatic ecosystem, increasing cyanobacterial cell densities also can pose a threat to human health (Smucker et al., 2021).

While only gaining general public awareness within the last two decades, references to “toxic algae” on small farm ponds leading to livestock sickness and water odor date back to the eighteenth century (U.S. National Office for Harmful Algal Blooms, 2024a,b). These events can alter the biochemical balance of aquatic ecosystems causing a range of ecological and social impacts such as severe illness or death in mammals (Figgatt et al., 2017), as well as fish kills (Zamor et al., 2014). If a HAB event is detected that reaches levels considered to be a risk to public health for water recreation (at or above 24 ug/L; World Health Organization, 2020), a no-use advisory may be issued for the waterbody and visitors may be turned away impacting the tourist industry and local communities. A survey on Lake Erie recreation showed that, on average, Lake Erie anglers canceled five separate trips in 2019 due to impacts of HABs on the lake leading to an estimated loss of $1.9–4.8 million dollars (Bennett et al., 2020). Bingham and Kinnell (2021) found that in the state of Ohio alone, tourism brings in an estimated $305 million annually, which is part of an estimated $12.9 billion industry spread out among the Great Lakes that can be affected by HABs.

Freshwater HABs are increasingly being researched in public health and environmental science in the United States (Hudnell, 2010; Sonak et al., 2018), but there is limited social science work to date on the management and human dimensions of freshwater HABs. Using social science research techniques can help build a platform for understanding the impact HABs have on recreation and communication needs. Armstrong et al. (2022) evaluated community awareness of HABs causes and solutions and found great variation in responses. The study concluded this variety necessitates disseminating additional information to communities regarding HABs risks as well as incorporating local opinions for solutions. Hardy et al. (2021) found outreach on recreational use was more common across the United States than on drinking water, and that funding is very limited for any outreach or monitoring programs. Boudreaux et al. (2022) found Lake Erie recreators were more wary to recreate after a bacterial alert was lifted as opposed to a HABs alert but did not address HABs management for these alerts. Van Dolah et al. (2015) found that a combination of short-term (HABs event treatment in a waterbody) and long-term approaches (nutrient management) are best for mitigating HABs from a recreational standpoint.

Though there is overall guidance by the United States Environmental Protection Agency (EPA) regarding HABs exposure limits in drinking water and recreation, there are currently no federal regulations for HABs monitoring in the United States (United States Environmental Protection Agency, 2019). Currently, U.S. states and tribes vary in their approaches to HAB monitoring, treatment, and communication. Many states only monitor for HABs in the summer months and have “emergency testing” capabilities if a possible bloom event has been reported (Ohio River Valley Water Sanitation Commission, 2021), while others have routine monitoring throughout a specific season for both HABs and water quality parameters related to HABs. According to Dodds et al. (2023), some states, such as the ones that are represented in this study, follow EPA guidelines for HABs recreational exposure thresholds as well as have “regular” monitoring whereas other states may be more or less strict. The United States is not alone in North America, as Rashidi et al. (2021) work in Canada concluded that without federal regulation, more populated provinces have more resources and money for HABs monitoring and management efforts than less populated provinces.

Our study aimed to identify how water managers and researchers perceive current HABs management across multiple regions of the United States, as well as their recommendations moving forward. This study helps fill the gap of understanding where management priorities lie for HABs as well as identifying possible areas of improvement across the United States.

## Methods

We conducted 28 semi-structured interviews to determine how key informants on HABs understand and perceive the current state and future priorities of HAB management they believe will help decrease the risk of future HAB events.

### Interview structure

Semi-structured interviews are used to explore specific topics with a wide array of questions (Hammarberg et al., 2016; Prokopy, 2010). The use of semi-structured questions allowed for a more fluid and holistic investigation of HABs monitoring and management that may not have been possible through a traditional survey or large group interview and created space for relevant follow-up questions (similar to insights in Burnham et al., 2016; Hammarberg et al., 2016; Prokopy, 2010). Some of the semi-structured interview questions were adapted from previous studies on perceptions on changing water quality to assess key informants’ perceptions of and knowledge about HABs (Deffner and Haase, 2018; Jacobs and Buijs, 2011) and others were developed specifically for this study. The interview questions focused on recreational issues with HABs. Information on concerns about HABs drinking water was not directly solicited in the questions nor was drinking water the intended focus of this study, although drinking water treatment was discussed by some Ohio and Mountain West key informants. The full set of interview questions can be found in the Supplementary material. All key informants were asked the same interview questions, with varying follow-up questions to clarify insights from the initial interview questions.

Interviews were conducted and recorded online using video meetings on Microsoft Teams during the spring and summer of 2022. To provide informed consent, all potential key informants were sent a form describing the voluntary nature of the interviews and the minimal risks and potential benefits of participation. All key informants that agreed to be interviewed answered all questions they were asked. This research was determined to be exempt from further review by the University of North Carolina at Chapel Hill Office of Human Research Ethics (Study #: 21–1840). Following completion of interviews, all personally identifiable information was removed for confidentiality.

### Key informants and regions of interest

Key informants are those with experience researching or managing for HABS. They were located primarily in New England (*n* = 12) and Ohio (*n* = 15), with four additional perspectives from the Mountain West of the United States (*n* = 4). There were 28 semi-structured interviews with 31 key-informant interview participants (hereafter “key informants”) due to three of the 28 interviews including two key informants each. Key informants were identified through purposive sampling. Purposive sampling intentionally selects potential key informants based on expertise or membership in the population of interest for the research (Palinkas et al., 2015). Some of the interview participants were identified through professional contacts and additional key informants were identified throughout the interview process from others interviewed. Most of the key informants were responsible for water management of multiple waterbodies rather than for a single waterbody, with the exception of key informants involved in decision making or water quality for Lake Harsha, Ohio. The key informants in this work hold important insights and roles for HABs management as past research documents a relatively high level of public trust in local water managers to protect public health (Voogd et al., 2021).

**FIGURE 1:**
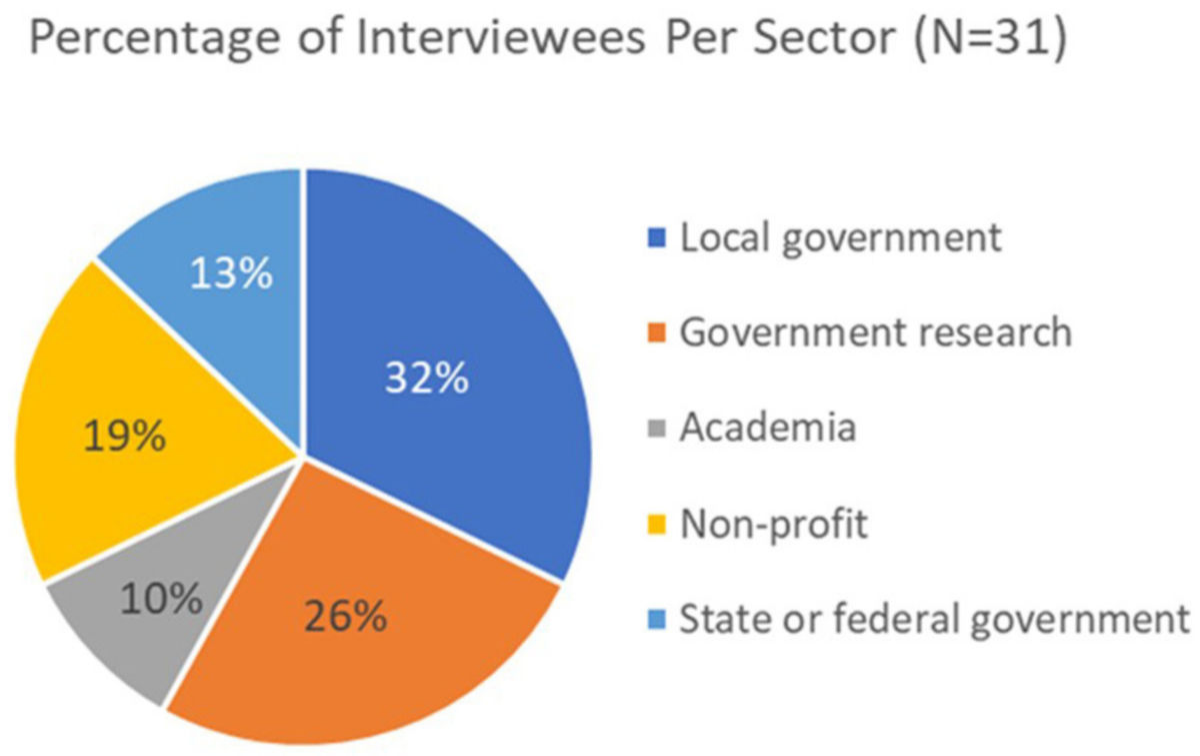
Pie chart depicting different sectors of the key informants who were interviewed. *N* = 31.

Because managing for HABs has many different aspects, including treatment, forecasting, monitoring, and communication, professional roles varied among those interviewed. Professions of the key informants included environmental planners, laboratory managers and analysts, water quality managers, water quality program directors, and general researchers, with the most common organizations of employment being government researchers and managers (Figure 1).

The key informants were based in New England (Rhode Island, New Hampshire, and Massachusetts, *n* = 12), Ohio (*n* = 15), and the Mountain West (*n* = 4) in the United States. The key informants focused upon waterbodies that varied greatly in their size with much of the focus in Ohio on the Ohio River and Lake Harsha (874 ha), lakes and ponds in and around National Parks in the Mountain West, and small ponds and lakes (generally <400 ha) in New England. The other waterbodies of interest included ponds and lakes of varying sizes used primarily for recreational purposes throughout the three regions (New England, Ohio, and the Mountain West). Waterbodies mentioned from key informants are considered at risk for HABs events or have had HABs advisories in the past. In this paper, the key informants are considered collectively, not regionally, except where specific geographic region is noted.

### NVivo transcription methods

Audio recordings of the interviews were imported from Microsoft Teams into NVivo (Version 12) software as mp4 files. NVivo was used to transcribe the audio to text for coding purposes. The transcriptions for all interviews were coded to identify and categorize relevant themes. Open coding assigned initial codes to the data based on its properties and axial coding drew connections among the initial codes to identify more specific themes or categories (Corbin and Strauss, 2015). All New England interviews were coded by one author and all Ohio and Mountain West interviews were coded by another author. An intercoder reliability assessment was performed between two team members using three interviews before analyzing all interviews for this study to ensure coding agreement was above 97% (similar to methods in Floress et al., 2017). Interview data were analyzed using a thematic analysis approach (Hsieh and Shannon, 2005). This qualitative data analysis approach identifies shared insights across key informants inductively rather than comparing data to anticipated findings (Mayring, 2014). Interviews were thematically coded using NVivo 12 qualitative coding software. Interview data were consolidated into nodes based on common phrases or topics mentioned by key informants. These nodes were then further broken into subthemes and analyzed for quotes that encompassed common answers to the questions. Common findings were then combined into sections by management aspect to highlight key findings (Fereday and Muir-Cochrane, 2006).

## Results and discussion

The key informants provided general insights about HABs management and described their perceptions of ongoing HABs management and priorities for throughout the HABs management process from prevention to communication (Table 1 and as broken into subsections of this section). While the interviewed water managers noted they “get lots of calls for algae and ponds” for non-harmful algae (often referred to as “nuisance” algae by the key informants) or aquatic plants, such as duckweed, present in a waterbody, their main concerns lay with cyanobacterial HABs. HABs were considered a higher concern for water quality management among all of those interviewed as opposed to nuisance algae because of the potential harm they pose to the ecosystem, economy, and public health. They noted the difficulty for the public and even water managers to determine the risk to humans and animals as harmful vs. non-harmful blooms cannot be determined just by appearance as toxin testing is required.

**FIGURE 2:**
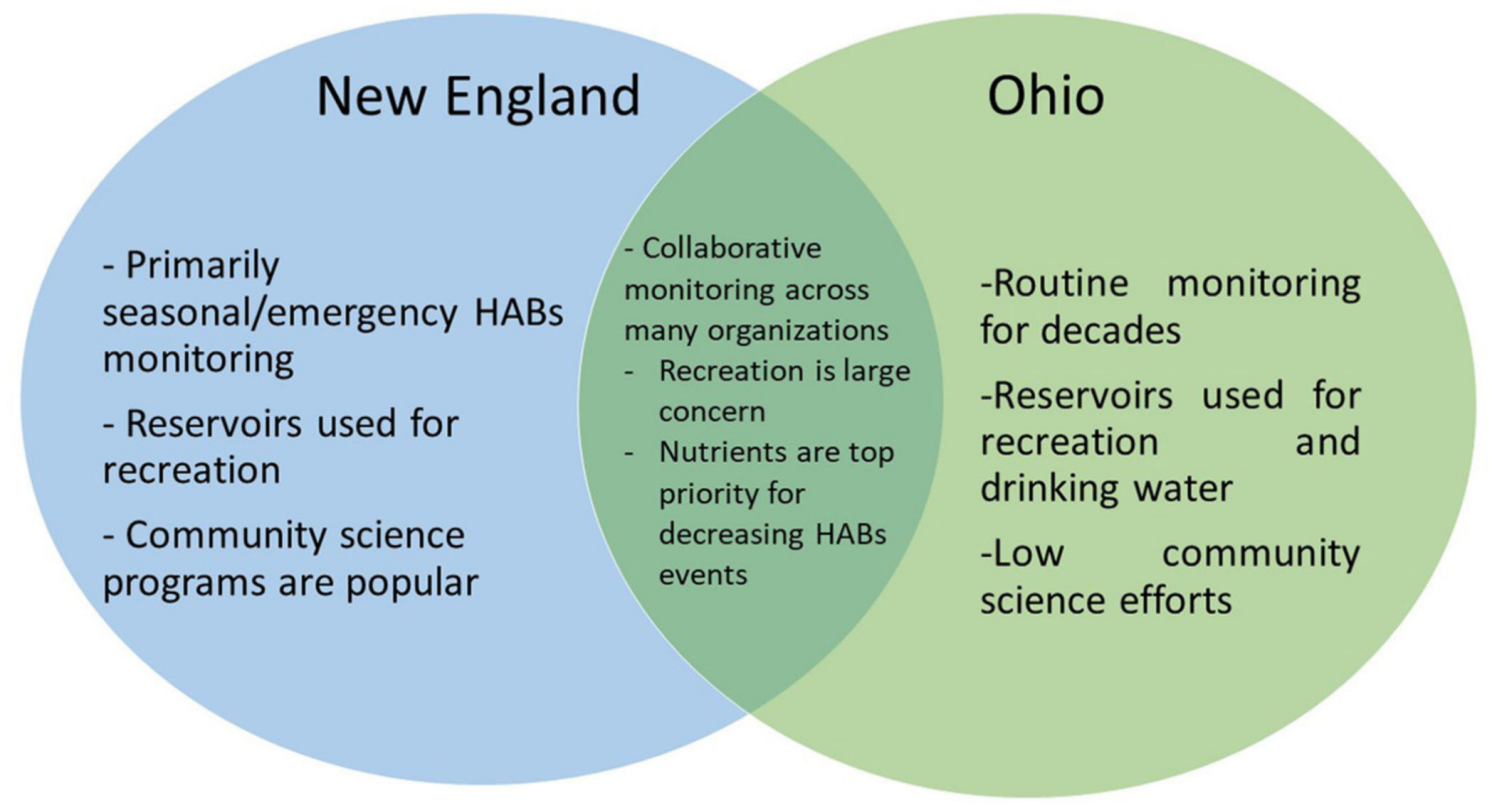
Differences in key informants’ perceptions across the regions.

The majority of the identified priorities were similar across the regions, such as making nutrient management a priority for preventing HABs events and recreational water use as a greater management concern than drinking water. There were also some differences in the they monitor, respond to, and educate about HABs across the regions (Figure 2). Some locations have increased their monitoring in the last decade and in other locations monitoring has remained consistent. While not explicitly asked about drinking water concerns related to HABs, almost all of the Ohio and Mountain West key informants discussed drinking water management, but key informants in New England did not. Additional differences are noted in context in the remaining subsections of this section.

When asked about potential negative impacts from increased HABs events, there were a variety of environmental, economic, social, and public health concerns mentioned. In terms of environmental consequences, key informants mentioned fish kills, harm to macroinvertebrates, decline in mussel diversity, degraded water quality, habitat degradation, scum, and odor. For economic impacts, key informants noted areas that rely on their affected waterbody for recreation and general tourism would suffer, especially if forced to cancel events that draw big crowds (e.g., rowing competitions on Lake Harsha, Ohio). If increasing HABs events were to occur, there would be a “short recreational window of access” during summer months for boating, swimming, and fishing. Some also mentioned the potential for decreasing house values as well as the general aesthetic and reputation of an area, similar to findings from past economic research across the country (e.g., Osseni et al., 2021; Wolf and Klaiber, 2017). Another economic concern from many key informants, regardless of occupational background, was increased spending for either HABs treatment in the source water or treatment in the drinking water plants to remove cyanotoxins and cell material. One key informant said that during a HABs event that occurred on the Ohio River in 2015, “Cincinnati Water Works spent six or seven thousand extra dollars a day just to make sure that [the drinking water] was safe for people to consume.”

Key informants noted potential public health concerns for more humans, pets, and livestock getting sick, believing children and older adults are the most at risk. Key informants made note that “dog deaths make the news,” specifically the case in Zion National Park in 2022. Those interviewed that interact with the public claimed that they do not hear too often of people getting sick and they hear far more stories about dogs getting sick or dying. These stories come from personal anecdotes of visitors to a waterbody of interest, news broadcasts, or social media. Those in the Ohio drinking water sector noted the significance of focusing events, for example “In 2014, due to the issues with Toledo having [cyano]toxin in their finished water, that got us more into the drinking water side of it.”

The key informants had mixed perceptions of whether HABs events are increasing. Some key informants perceived an overall increase in HABs events in the last decade, ranging from perceived increases in no-swim advisories due to HABs events, like those in Lake Harsha from 2016 to 2021 (Ohio Beach Water Quality Monitoring, 2024), to a New England key informant who noted a “7-fold increase in algal blooms reports.” Other key informants from New England found it difficult to determine if there had been an increase in HABs events or if they are simply more aware of them due to an increase in monitoring despite recent research that has identified global patterns of HABs increasing (e.g., Fang et al., 2022; Griffith and Gobler, 2019; and Wells et al., 2015).

**FIGURE 3:**
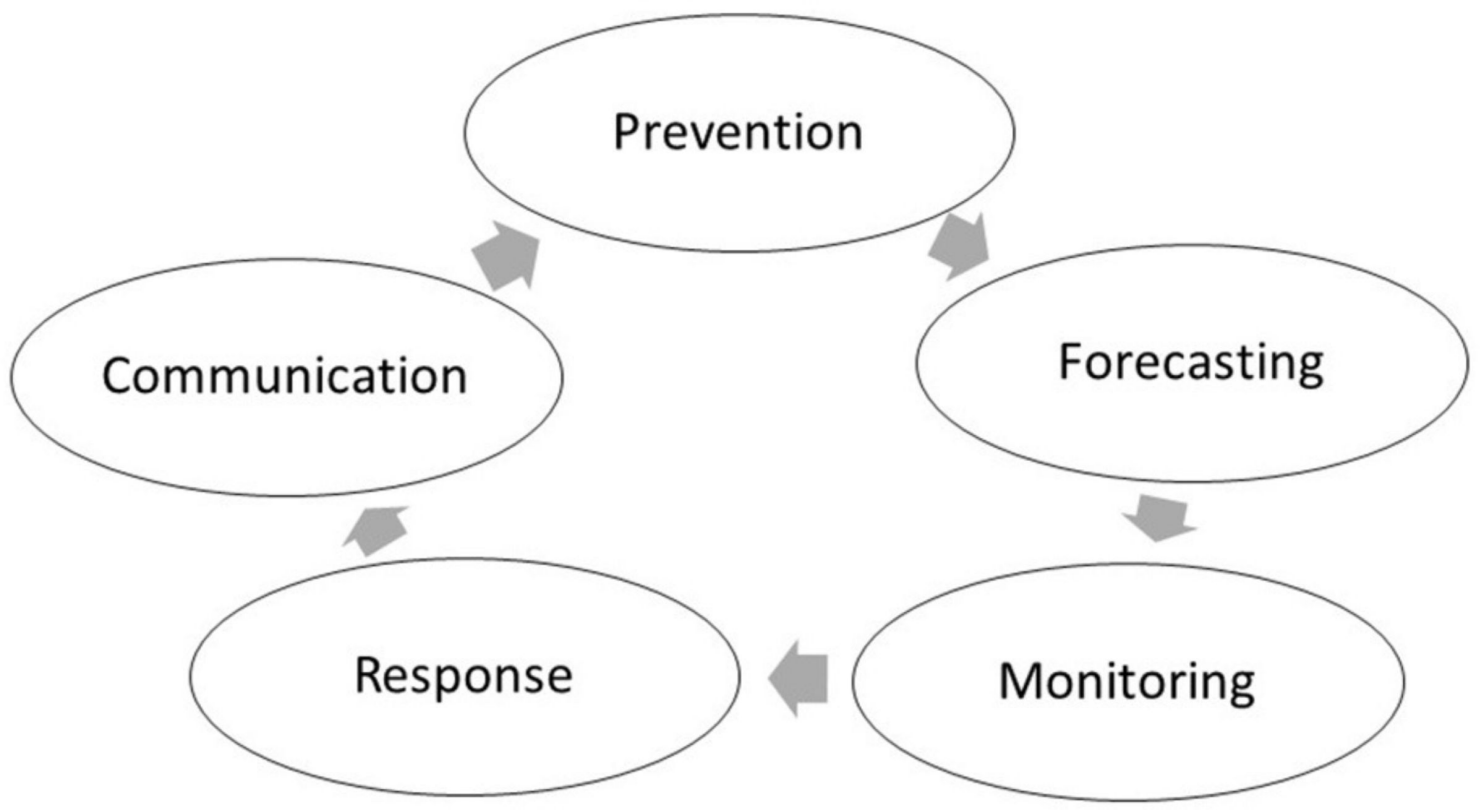
Insights (current efforts, issues, and needs) of HABs management.

Key informants shared current perceptions and issues, as well as recommendations for future efforts to minimize negative impacts of HABs. These perspectives fell into the major themes of prevention of HABs events, forecasting and monitoring for HABs, efficient responses to HABs events, and communicating HABs risks (Table 1). Below, we discuss the findings based on these themes in order beginning with prevention (Figure 3).

### Preventing harmful algal blooms

Nutrient overload and management were considered the top water quality concerns among key informants regardless of whether they work on nutrients or not, with HABs being the next most popular answer. One key informant said, “nutrients are a huge factor, *of course!*” Another said, “reducing nutrients is the best solution once a body of water has harmful algal blooms in it.” The emphasis on reducing nutrient loading for HABs management is similar to the findings from past HABs research (Green et al., 2023; Griffith and Gobler, 2019; Bennett et al., 2020). Nutrient loading concerns of the managers across the regions reflected differences in land use between Ohio and New England. Ohio has a large amount of agricultural production with related nutrient loading sourced from the use of fertilizer causing runoff laden with excess nutrients. Key informants in New England also cited nutrients as a main contributor to HAB activity, but due to different land use in the region, wastewater runoff was the primary nutrient source rather than agriculture. In the Mountain West, land is used for a mix of agricultural production, but a large portion is forested and protected for recreation. Regardless of region, key informants in all of the interviews mentioned issues with nutrients as a main concern for HABs management.

Those interviewed described HAB prevention as a joint effort among the general public, farmers, homeowners, industries, and researchers. Though most key informants were neutral in terms of their opinions on treating HABs, one drinking water manager did note they are against long-term chemical treatments because they do not treat the source of the problem. That manager would rather see the prevention of the drivers of HABs, specifically decreasing nutrient inputs and concentrations of legacy nutrients already in a waterbody by way of nutrient binding or lake dredging. Key informants mentioned individual actions homeowners and farmers can take to prevent HAB events, such as upgrading aging septic systems, building vegetation buffers, and decreased fertilizer use. The key informants were aware that nutrient management is “not a quick fix or an easy fix” due to the slow impact and challenges in implementing these changes at the individual property level. Other suggested local solutions were green infrastructure, aeration of a waterbody, or physically changing the landscape by constructing wetlands across tributaries into a body of water. Rather than having one nutrient reduction method be a panacea, many key informants identified the need for multiple approaches.

Some key informants argued that mitigating effects of climate change could have lasting effects on HABs activity, especially preventing or reversing increased water temperature and decreasing rainfall. Key informants noted that while we have some control over our climate impact, that’s a “bigger and broader” issue. One key informant said, “climate change is a national and worldwide problem that we have with carbon emissions... Whereas, nutrient runoff we can address a little more locally. Sometimes the problem just seems enormous.”

While key informants identified the global and regional concerns related to nutrients and climate change, they focused on more localized solutions, with an overall goal to minimize anthropogenic contributions to increased HABs activity. One key informant noted, “We are doing so much in our watershed. Trying to reduce nutrient loading can help us address water quality concerns, primarily being harmful algal blooms, but eutrophication in general, and also the impacts on biological water quality.” Prevention methods recommended range from farmers implementing best management practices on their land, to local governments creating wetlands or riparian corridors around a lake to individual households using less fertilizer that will runoff into a waterbody. One participant explained the consensus among key informants that, “HABs aren’t going anywhere anytime soon,” but there are things that can be done to possibly help prevent them from being so frequent or severe. The prevention methods mentioned (either individually or organizationally implemented) could not only help reduce potential HABs events, but also address the overall nutrient-related water quality concerns shared among those interviewed. Several informants highlighted that nutrient management plans need to be tailored to the specific nutrient sources and the sizes of different waterbodies.

### Forecasting harmful algal blooms

Forecasting is a relatively new tool in understanding and communicating freshwater HABs that may be beneficial to many groups of researchers, stakeholders, and the public if used correctly. Forecasting can be defined as the prediction or estimation of future events accompanied by an estimation of uncertainty around the prediction and explaining the rationale for the prediction (Scavia et al., 2021). Forecasting tools are being tested to identify where and when a HABs event will potentially occur in a body of water. HABs forecasting models are similar to weather models in that they show current conditions, can predict conditions up to 5 days in advance and expectations for a full season (generally the summer), and try to determine the movement of a bloom within a waterbody (Gill et al., 2018).

The overall reaction from those interviewed was that forecasting has potential as a tool for water managers and the public and that forecasting is the future of managing HABs more efficiently. As of right now, key informants identified the use of water quality data as their current practice as opposed to using any type of forecasting. “Beyond solving it and stopping [HABs], it’s notifying when it’s coming. It’s the most important thing that you can let the word out... There’s all sorts of different things you can use to combat it and deploying those at the right time is critical to success.” Forecasting allows public health officials to be aware of possible hazards and, if used correctly, can help prepare drinking water and water recreation managers for potential changes in operations. It also would allow for advisories to be posted and the public to be notified and make informed decisions about putting themselves at risk during a HABs event. Another key informant claimed “forecasting is critical, because if we aren’t able to forecast in advance, then by the time there’s a bloom, it’s too late. People have already been in the pond for a week or 2 or days where they may not have known.”

Other key informants were concerned that forecasting is not sufficiently advanced. “Environmental conditions and the watershed circumstances can change a lot,” noted one key informant when asked about the role forecasting can play in freshwater management. “You have to keep [the models] up to date so that they continue to be representative. For the most part we find that we don’t get very good predictions and that makes sense to me, because we don’t really capture all of the ecology that goes into bloom formation, community structure, and toxin production. You can’t produce good models if the underlying mechanistic features [of HABs] are not incorporated.” Another key informant said, “I personally don’t see the benefit in it. I think it’s cool science... but I don’t know if it helps anybody. In and of itself, maybe it’s not that useful yet. I can see how it can be built upon.” This key informant also argued that saying a bloom is coming is not enough—knowing exactly when it will happen is necessary for being proactive. Many of the key informants noted that as technology with water quality monitoring and satellite imagery improves, it is important to update forecasting models.

### Monitoring harmful algal blooms

The interviews revealed considerable differences in monitoring among regions, within states in a region, and within individual states (similar to findings of Dodds et al., 2023). The inconsistency included differences in monitoring methods, duration, and frequency. One key informant noted, “how variable the states are and how they monitor for HABs, that kind of scares me a little bit because I think about all the potential interactions that the public will have with the waterbody and not know.” One key informant working in the Ohio River Valley noted Ohio’s routine monitoring has shown HAB activity as far back as the 1990’s. Monitoring efforts have varied or changed over time from documenting cell density to documenting toxin concentrations to better quantify indicators of risk to human health. Ohio currently has not only emergency monitoring, but also long-term routine monitoring outside of the traditional HABs season for some waterbodies (Ohio River Valley Water Sanitation Commission, 2021). This monitoring occurs in waterbodies like the Ohio River, the Lake Erie basin, Grand Lake St. Mary’s, and Lake Harsha—all locations that are used heavily for drinking water reservoirs and recreational activity, as well as having seasonal HABs events. Ohio key informants perceived that overall monitoring for HABs over the last decade has not increased, but rather remained consistent.

Other areas have focused efforts on HABs more recently. One key informant in the Mountain West mentioned, “We started in 2016... prior to that, we did not monitor at all.” For example, Colorado has a set budget given to regional labs based on how many samples each lab plans to run throughout the summer months. Some states in New England operate similarly, with select waterbodies of interest receiving as-needed HABs monitoring in the summer. Some monitoring efforts are rapidly increasing due to public interest. One key informant in New England said, “we’ve only recently ramped up.... hopefully this year [2022] we get more than twice that.” The key informants placed an emphasis on monitoring recreational locations to ensure the safety of visitors. This means that “emergency” or as-needed monitoring occurs—generally during peak visitation season—when a visitor or park ranger reports what could be a potential bloom. Key informants often said they do patrols of a park, specifically a “quick visual inspection” if someone called in a possible bloom. This then triggered a sample collection and lab screening for toxins. Though a monitoring program that could yield a no-visit advisory may lead to revenue loss, informants also saw monitoring as being in the recreational areas’ best interest to protect the public health and safety of their visitors. New England key informants noted an increase in monitoring efforts over the last 5 years, with an emphasis on summer month monitoring for “problem” ponds. The perceived increase in monitoring caused some key informants to question if HABs events are increasing or if the events are just being captured better. This supports the need for clear and consistent monitoring over time to better detect impacts from climate change or other environmental changes.

The scale, scope, and complexity of HABs monitoring was noted by key informants as a significant challenge that has been addressed through collaborative efforts. HABs monitoring efforts were identified as highly collaborative in the studied regions and tended to focus on specific ponds or lakes. A program may be dedicated to monitoring a certain waterbody, but the work is often shared among state and local government groups as well as community science efforts or university research groups. In Ohio, for example, the Ohio EPA and ORSANCO are proactive in HABs monitoring. In New Hampshire, there is a prolific community-based cyanobacteria monitoring program through the University of New Hampshire. Key informants often mentioned that with water samples, it is common for one group to collect the sample and another to perform the toxicity analysis. Key informants involved in monitoring efforts agreed that collaborative programs can be beneficial for decision-making and spreading the work so one organization does not bear the full monitoring workload. Between those monitoring in the field, those testing samples in the lab, and those that manage a waterbody, identifying and treating a bloom when toxic or hazardous, monitoring is a multidisciplinary feat. Key informants expressed that while funding is often difficult to secure for long periods of time, and comes from many different sources, the shared burden of HABs efforts makes it easier for labs to focus on other projects if needed.

Key informants identified changes in how much funding is allocated to different organizations for monitoring efforts. For example, one key informant with jurisdiction over Ohio and Kentucky water quality monitoring claimed that they used to do more routine monitoring throughout the Midwest before the state programs became more prominent, and the responsibilities shifted. For special cases or projects, key informants mentioned additional funding can come from the state or the Ohio Department of Higher Education or NOAA. Most recently, funding for Ohio freshwater HABs has come from the U.S. Army Corps of Engineers to help detect and monitor HABs in Ohio, specifically the Maumee River (Billau, 2023). Key informants with a larger regional jurisdiction said their offices also have the capability to fund state monitoring programs. Overall, funding in Ohio comes from a variety of sources and there seems to even be an increase in money awarded from these various programs to help continue or enhance current monitoring practices. Key informants from Ohio did not touch on funding specifically being a hindrance to their monitoring efforts but mentioned the different programs listed above as sources for funding.

Funding for both monitoring and toxicity testing are of concern for those managers interviewed in New England. “It’s really expensive,” one key informant noted. “One thing that [monitoring] has going for it is that [HABs are] such a hot topic right now.” Another key informant noted that priorities for monitoring are established based on the level of funding. “I know that we’re not effectively spending money because it’s split in so many different ways and it does not come back to the people who need it.” This is similar to the findings of Margerum and Robinson (2015), who found that collaborative efforts can make it more difficult to efficiently and fairly allocate funding for monitoring, especially if the process for decision-making and funding varies across multiple organizations.

In New England, informants noted that the ponds that are known to be the worst for HABs activity are addressed first as opposed to establishing a routine cover-all procedure. One key informant noted that, “We have a set of sites that are monitored routinely every 2-to-3 weeks, depending on funding... then there is additional monitoring and follow-up monitoring that happens at sites that do have problems. Our team has been told to talk about cyanobacteria activities and not call it a program because we don’t have stable funding.” Mountain West key informants also shared the same sentiment that some locations are more likely to experience more HABs events and thus are higher priority for monitoring, however lab funding can still change year to year as far as their capacity for monitoring and how many samples they are allowed to run.

Overall, an increase in routine monitoring was seen as needed in the many locations where people recreate and/or water is used for drinking despite the financial and workload challenges. Key informants supported routine monitoring because it can help create a database for researchers and government institutions to access current and past data to make inferences about HABs activity and general water quality. They noted that without monitoring, it is impossible to know trends in a waterbody of interest and to prevent or treat HABs. In pushing for routine monitoring, key informants noted that associated funding must also be reliable to make long-term positive change with monitoring practices across the regions.

### Responding to harmful algal bloom events

When HABs prevention is not implemented or does not work, water managers have to respond to HABs events as they are occurring or after. The key informants described a number of surface water treatments for mitigating HAB events, including chemical treatments, ultrasonic waves, aeration, and constructed wetlands. This lack of a single treatment solution adds to the complexity of managing for HABs. The key informants who discussed drinking water concerns noted that existing drinking water treatment options would take care of HABs concerns. They believed their systems to be well-equipped to handle anything that reaches their intake since, “[we] have the same strategy [for HABs] as we do for all our problems... the treatment method is the same.”

While there are a number of surface water treatments being used or piloted, the key informants had a strong preference for preventing the HABs events rather than treating for them. One key informant said, “but that does sort of just treat the symptoms, not the actual source of the problem, and so nutrient levels would still be high.” The key informants noted that surface water treatments do not always work, either due to efficacy or minimal data supporting an improved water quality post-treatment [similar to findings in Anantapantula and Wilson (2023)]. For example, one key informant said chemical treatment solutions are “too often applied as the only default. And they’re not very effective, cause a lot of ecological damage, and aren’t long term.” Because of the complexity of HABs both in the long-term and short-term, several key informants believed that a combination of methods must be used to manage for HABs.

There was an interest in more timely treatment of HAB events to allow for fewer advisories or closings, which can benefit local communities culturally and economically. Treating a HAB event may allow for a swifter return to normal water use and avoid disrupting communities and visitors long term, however, may not be possible. As one key informant said, “I think people get frustrated that these are things that can’t be fixed overnight... it’s not an easy fix and everybody’s kind of contributing to the problem. And sometimes it’s hard to hear.”

The disaggregated management of HABs was identified by key informants as a challenge in responding to HABs events. Key informants identified that it is difficult because different organizations focus on different aspects of management. To accomplish this well-rounded understanding of risk and current work, several key informants identified a need for a “central agency incorporating all of the work that’s going on.” The key informants recommended that a centralized hub of research would support local educational programs and compile water quality data in one place, rather than having to navigate multiple websites for this information. One key informant noted that “aggregating information.... would be the easiest way to jump over hurdles of credibility or information.” Another key informant described this as “it’s like every agency builds their own. It goes off in their own direction and it’d be nice to circle back around, have that connection again.” Key informants also noted that universities in particular have their own research and foci compared to water management agencies. Having a centralized, coordinated effort to better identify current research gaps and project efforts would help alleviate the gaps in knowledge between multiple groups working on HABs.

### Communicating harmful algal blooms

Efforts to engage and communicate with the public about HABs ranged from intensive community-science monitoring programs to more passive signs and brochures and were identified as improved by most of the key informants.

Key informants in Ohio noted many educational programs dedicated to water quality for a range of target audiences but stated that these programs do not focus specifically on HABs. The educational programs address general water quality concerning the Ohio River and best management practices regarding nutrient management. One key informant noted a water quality program focused on the Ohio River “probably over 10 years ago now” did mention potential causes of HABs and was generally well-received. However, they could not recall any current programs that do this. Ohio key informants overall expressed interest in additional HABs education being taught but could not recall any plans to do so soon.

Some differences in communication modes were regional, with New England having more programs and opportunities for the community to be involved in science and help with HABs in their area, including Providence Stormwater Innovation Center, University of Rhode Island’s Watershed Watch, and national EPA efforts such as the Cyanobacteria Assessment Network Application (CyAN App), cyanoScope, and bloomWatch (https://cyanos.org/cyanoscope/). These programs provide volunteer and monitoring opportunities for local involvement from collecting water samples to taxonomic identification. The programs in New England grew over the last few years increasing the volunteers and ponds of interest. One key informant said, “we had fifty volunteers in this citizen science program and actually had to turn away people, so that was pretty exciting.” They also have education programs tailored to young students and that teach about general water quality and nutrient management, including Save The Bay, University of Rhode Island Cooperative Extension, and the Cyanobacteria Monitoring Collaborative.

Key informants across the included regions perceived the general public’s awareness of harmful algal blooms as increasing, but only at a basic level. Ohio key informants mentioned Toledo, Lake Erie, and Grand Lake St. Marys as the key locations whose HABs events sparked interest. Others cited Florida, specifically Lake Okeechobee, and the increased media coverage as leading to increased awareness. Many key informants believed the public may know the term “harmful algal bloom” means something bad in the water, but do not know what to look for, where to find information, or the consequences of exposure. One key informant compared the phenomenon of HABs to that of mercury in fish in that the public is slowly becoming more informed of HABs and their risks, similar to how people became used to public health warnings about mercury. This key informant claimed “when [mercury concerns] first started, it was like, ‘What are you talking about?’ I think that’s kind of where we’re at [with HABs].”

The key informants who interact with recreators regularly at waterbodies noted that people are asking more questions about HABs. They identified those involved in water recreation sports (boating, paddling, swimming, etc.) who use waterbodies that are affected by HABs or those who are involved with community science programs have more concern or awareness of good vs. poor water quality than an average beachgoer. Recreationists seemed concerned about water quality when they see someone “official” walking around the park and want to ensure their safety before entering the water. Recreators at the boat ramps who noticed park employees or researchers have occasionally asked if the water is safe to be in, but did not always name HABs directly as a potential issue. They were more so curious about what the employee was doing at the park. While they may be increasingly curious about HABs and mildly more familiar with the term, key informants perceived continued low risk assessment among recreators.

The key informants that interact with recreators noted they are much more likely to be asked about water quality in person at a recreation site as opposed to a phone call or email to their office, highlighting the importance of educating on site for those most likely to be affected. Additionally, some of those interviewed felt it was important to communicate to decision makers the need for funding for HABs research and to create additional monitoring programs and incentives for best management practices across the country, especially in states that currently do not have one. According to those interviewed, there is an upward trend of basic knowledge and understanding that HABs can be harmful, but more work should still be done. Even with this basic understanding, most key informants agreed that more HABs-focused education programs and regulations should be implemented to ensure better safety of those around a waterbody prone to HABs.

## Conclusion

This paper presents various perspectives on the current challenges in freshwater HABs management and provides considerations for future HABs management. HABs are traditionally seen as an ecological problem to be solved in the lab or the field. The interviews pointed to the benefits and challenges of emphasizing cross-disciplinary and holistic approaches to managing HABs (similar to Dewulf et al., 2007; Armstrong et al., 2022) while bringing in the needed human aspect through key informant perspectives to identify needs for managing HABs.

To improve HABs management, the key informants emphasized the importance of continuing to consider nutrient reduction to prevent HABs events and improving communication for HABs. While nutrient management is a long-term issue, it is preferred as a preventative method by key informants for HABs events as opposed to reactive treatment of a HABs event. This is because treatments are considered short-term fixes to an ongoing problem. To address the issue of human and animal health risk, a public-centered recommendation was the need to continue awareness of HABs such that waterbody users know not only what HABs are but the risks they pose to public health and recreation.

As mentioned by many key informants, there is no quick fix to the many factors driving HABs, the risks they pose, or the methods of treatment once they occur. Recommendations across the regions were to highlight the importance of and need for an increase in routine monitoring to allow for accurate identification of and prompt response to HABs. The key informants also identified the value of combining various nutrient management practices to potentially reduce the risk of HABs to the environment and people (Table 1).

While this study provides insights across three regions of the United States, additional interviews with additional key informants from the other regions and countries could create a more holistic view of management practices and identify new transferable best practices for management and risk communication. There is also a need for future work to broaden the understanding of human dimensions. For example, this research was framed around recreation-related HABs concerns (which were identified as the primary concern), but many of the interview informants still brought up drinking water considerations, indicating the importance of future work focused also on drinking water. The qualitative insights gathered in this study on HABs management provide useful information for cross-project comparisons. Future projects could use the data acquired in this study to create nuance in questions or methodology used for quantitative analysis.

Overall, recreational use of freshwater lakes, ponds, rivers, and other waterbodies provides important social, health, and economic value to local communities and visitors. As these waterbodies are increasingly impacted by HABs, management to protect human health and the environment and to continue recreational use of valued waterbodies will need to consider a complex set of decisions and efforts from prevention through communication. While the key informants identified opportunities for improving management, they also noted considerable progress has been made in non-point source management of nutrients, forecasting potential waterbodies of concern, monitoring HABs affected waterbodies, treating blooms that have occurred, and communicating potential risks. Collaborating across practitioners, managers, and researchers throughout the management process to build upon the existing efforts and meet identified needs can better protect these important resources.

## Supplementary Material

Supplement1

## Figures and Tables

**TABLE 1:** Current management practices, issues, and goals moving forward as defined by key informants.

	Prevention	Forecasting	Monitoring	Responding	Communication
Current management	• Preference by many for prevention rather than treatment • Nutrient management is the primary concern • Range of best management practices in place	• Use collected water quality data to predict HABs, not forecasting • Not being actively used.	• Routine and emergency monitoring are common for some waterbodies, but insufficient at scale	• Various HABs treatments (e.g., chemical, physical) are used in HABs-affected waterbodies • Beaches closed to recreational use • Drinking water treatment	• Likely an increase in basic awareness from the public • Range of communication efforts being used
Current issues	• Nutrient management is a large-scale problem. • Climate change affects HABs and could increase HABs events	• Shows promise, but needs advancement	• Funding changes monitoring priorities • Emergency monitoring requires quick turnaround • Many sites remain unmonitored	• Treatments are costly • Timely response is difficult • Not enough research on which source water treatments are most effective	• Communication efforts not consistently implemented • Public needs deeper understanding on the risks of exposure
Moving forward	• Continue and increase long-term management of nutrient loads (N&P) into waterbody	• Adapt forecasting models using more ecological data • Use forecasting for day-to-day use, not just seasonal trends	• Ensure consistent funding for routine monitoring • Centralize data • More routine monitoring	• Combine preventative and reactive measures • More research on treatments	• Need better collaboration and data sharing among groups managing or studying HABs • Develop consistent risk communication materials

Main topics covered include prevention of HABs events, forecasting HABs, monitoring water quality for HABs, responding to HABs events, and communicating about HABs with the public.

## Data Availability

The raw data supporting the conclusions of this article will be made available by the authors, without undue reservation.

## References

[R1] Anantapantula SS, and Wilson AE (2023). Most treatments to control freshwater algal blooms are not effective: meta-analysis of field experiments. Water Res. 243:120342. doi: 10.1016/j.watres.2023.120342 37544109

[R2] Armstrong A, Stedman RC, Sweet S, and Hairston N. (2022). What causes harmful algal blooms? A case study of causal attributions and conflict in a lakeshore community. Environ. Manag. 21:9. doi: 10.1007/s00267-021-01581-9 35031890

[R3] Bennett A, Benitez A, Litwin E, and Towns G. (2020). An Assessment of the Impact of Algal Blooms on the Economic Contributions of Recreational Angling in the Michigan Waters of Lake Erie. Available at: https://mucc.org/wp-content/uploads/2022/10/HABs-Economic-Impact-Report-to-MUCC-2.pdf (accessed August 7, 2023).

[R4] Billau C. (2023). Utoledo Awarded $1.5 Million to Fight Algal Blooms in Reservoirs, Rivers used for Public Water Supply. Utoledo News, UToledo News, The University of Toledo News. Available at: https://news.utoledo.edu/index.php/04_17_2023/utoledo-awarded-1-5-million-to-fight-algal-blooms-in-reservoirs-rivers-used-for-public-water-supply (accessed January 4, 2024).

[R5] Bingham M, and Kinnell J. (2021). The tourism impacts of Lake Erie hazardous algal blooms. Inland Wat. Dyn. Ecol. 2021:93625. doi: 10.5772/intechopen.93625

[R6] Boudreaux G, Lupi F, Sohngen BL, and Xu A. (2022). Measuring beachgoer preferences for avoiding harmful algal blooms and bacterial warnings. SSRN Electr. J. 2022:4019271. doi: 10.2139/ssrn.4019271

[R7] Burnham M, Ma Z, Endter-Wada J, and Bardsley T. (2016). Water management decision making in the face of multiple forms of uncertainty and risk. J. Water Resour. Assoc. 52, 1366–1384. doi: 10.1111/1752-1688.12459

[R8] Center for Disease Control and Prevention (2018). Freshwater Environments. Harmful Algal Blooms. CDC. Available at: https://www.cdc.gov/habs/illness-symptoms-freshwater.html (accessed December 20, 2023).

[R9] Cheung MY, Liang S, and Lee J. (2013). Toxin-producing cyanobacteria in freshwater: a review of the problems, impact on drinking water safety, and efforts for protecting public health. J. Microbiol. 51, 1–10. doi: 10.1007/s12275-013-2549-3 23456705

[R10] Corbin J, and Strauss A. (2015). Basics of Qualitative Research: Techniques and Procedures for Developing Grounded Theory, 4th Edn. Los Angeles, CA: Sage.

[R11] Deffner J, and Haase A. (2018). The societal relevance of river restoration. Ecol. Soc. 23:230435. doi: 10.5751/ES-10530-230435

[R12] Dewulf A, François G, Pahl-Wostl C, and Taillieu T. (2007). A framing approach to cross-disciplinary research collaboration: experiences from a large-scale research project on adaptive water management. Ecol. Soc. 12:214. doi: 10.5751/ES-02142-120214

[R13] Dodds WK, Bonjour SM, Fisher M, Krueger LJ, Pfaff PJ, Raihan MA, (2023). A novel index reveals disconnects between recreational harmful algal bloom exposure risks and responses among U.S. states. J. Am. Water Resour. Assoc. 60, 273–286. doi: 10.1111/1752-1688.13181

[R14] Fang C, Song K, Paerl HW, Pierre-Andre J, Wen Z, Liu G, (2022). Global divergent trends of algal blooms detected by satellite during 1982–2018. Glob. Change Biol. 28, 2327–2340. doi: 10.1111/gcb.16077 34995391

[R15] Fereday J, and Muir-Cochrane E. (2006). Demonstrating rigor using thematic analysis: a hybrid approach of inductive and deductive coding and theme development. Int. J. Qualit. Methods 5, 80–92. doi: 10.1177/160940690600500107

[R16] Figgatt M, Hyde J, Dziewulski D, Wiegert E, Kishbaugh S, Zelin G, (2017). Harmful algal bloom-associated illnesses in humans and dogs identified through a pilot surveillance system—New York, 2015. Morbid. Mortal. Weekly Rep. 66, 1182–1184. doi: 10.15585/mmwr.mm6643a5 PMC5689215 29095808

[R17] Floress K, Kolozsvary MB, and Mangun J. (2017). Expert perceptions of approaches to protecting isolated wetlands in the northeastern United States. J. Am. Water Resour. Assoc. 53, 1048–1061. doi: 10.1111/1752-1688.12553

[R18] Gill D, Rowe M, and Joshi SJ (2018). Fishing in greener waters: understanding the impact of harmful algal blooms on Lake Erie anglers and the potential for adoption of a forecast model. J. Environ. Manag. 227, 248–255. doi: 10.1016/j.jenvman.2018.08.074 30199720

[R19] Gobler CJ (2019). Climate change and harmful algal blooms: insights and perspective. Harmful Algae 91:101731. doi: 10.1016/j.hal.2019.101731 32057341

[R20] Green SR, Waldmann Rosenbaum C, Hughes S, Wu X, Dusicska E, Sun K, (2023). Nutrient management in Lake Erie: evaluating stakeholder values, attitudes, and policy preferences. J. Great Lakes Res. 49, 746–756. doi: 10.1016/j.jglr.2023.03.007

[R21] Griffith AW, and Gobler CJ (2019). Harmful algal blooms: a climate change co-stressor in marine and freshwater ecosystems. Harmful Algae 91:8. doi: 10.1016/j.hal.2019.03.008 32057338

[R22] Hammarberg K, Kirkman M, and De Lacey S. (2016). Qualitative research methods: when to use them and how to judge them. Hum. Reproduct. 31, 498–501. doi: 10.1093/humrep/dev334 26759142

[R23] Hardy FJ, Preece EP, and Backer LC (2021). Status of state cyanoHAB outreach and monitoring efforts, United States. Lake Reserv. Manag. 37, 246–260. doi: 10.1080/10402381.2020.1863530 35928550 PMC9348555

[R24] Heil CA, and Muni-Morgan AL (2021). Florida’s Harmful Algal Bloom (HAB) problem: escalating risks to human, environmental and economic health with climate change. Front. Ecol. Evol. 9:80. doi: 10.3389/fevo.2021.646080

[R25] Hsieh H-F, and Shannon SE (2005). Three approaches to qualitative content analysis. Qualit. Health Res. 15, 1277–1288. doi: 10.1177/1049732305276687 16204405

[R26] Hudnell HK (2010). The state of U.S. freshwater harmful algal blooms assessments, policy and legislation. Toxicon 55, 1024–1034. doi: 10.1016/j.toxicon.2009.07.021 19646465

[R27] Jacobs MH, and Buijs AE (2011). Understanding stakeholders’ attitudes toward water management interventions: role of place meanings. Water Resour. Res. 47:8366. doi: 10.1029/2009WR008366

[R28] Malone TC, and Newton A. (2020). The globalization of cultural eutrophication in the coastal ocean: causes and consequences. Front. Mar. Sci. 7:670. doi: 10.3389/fmars.2020.00670

[R29] Margerum RD, and Robinson CJ (2015). Collaborative partnerships and the challenges for sustainable water management. Curr. Opin. Environ. Sustainabil. 12, 53–58. doi: 10.1016/j.cosust.2014.09.003

[R30] Mayring (2014). Qualitative Content Analysis—SSOAR, Qualitative Content Analysis: Theoretical Foundation, Basic Procedures and Software Solution. Available at: https://www.ssoar.info/ssoar/bitstream/handle/document/39517/ssoar-2014-mayring-Qualitative_content_analysis_theoretical_foundation.pdf (accessed August 01, 2024).

[R31] Ohio Beach Water Quality Monitoring (2024). Beachguard. Available at: https://publicapps.odh.ohio.gov/beachguardpublic/beaches/191/samplings (accessed January 4, 2024).

[R32] Ohio River Valley Water Sanitation Commission (2021). Harmful Algae Bloom Monitoring, Response and Communication Plan Final. Available at: https://www.orsanco.org/wp-content/uploads/2021/02/FINAL-2021-HAB-Monitoring-and-Response-Plan.pdf (accessed January 4, 2024).

[R33] Olson NE, Boaggio K, Rice B, Foley KM, and LeDuc SD (2023). Wildfires in the western United States are mobilizing PM2.5-associated nutrients and may be contributing to downwind cyanobacteria blooms. Environ. Sci. 25, 1049–1066. doi: 10.1039/D3EM00042G PMC10585592 37232758

[R34] Osseni AF, Bareille F, and Dupraz (2021). Hedonic valuation of harmful algal bloom pollution: why econometrics matters? Land Use Pol. 107:104283. doi: 10.1016/j.landusepol.2019.104283

[R35] Paerl HW, Gardner WS, Havens KE, Joyner AR, McCarthy MJ, Newell SE, (2016). Mitigating cyanobacterial harmful algal blooms in aquatic ecosystems impacted by climate change and anthropogenic nutrients. Harmful Algae 54, 213–222. doi: 10.1016/j.hal.2015.09.009 28073478

[R36] Palinkas LA, Horwitz SM, Green CA, Wisdom JP, Duan N, and Hoagwood K. (2015). Purposeful sampling for qualitative data collection and analysis in mixed method implementation research. Admin. Pol. Mental Health Mental Health Serv. Res. 42, 533–544. doi: 10.1007/s10488-013-0528-y PMC4012002 24193818

[R37] Prokopy LS (2010). Agricultural human dimensions research: the role of qualitative research methods. J. Soil Water Conserv. 66:9A12A. doi: 10.2489/jswc.66.1.9A

[R38] Rashidi H, Baulch H, Gill A, Bharadwaj L, and Bradford L. (2021). Monitoring, managing, and communicating risk of harmful algal blooms (HABs) in recreational resources across Canada. Environ. Health Insights 15:117863022110144. doi: 10.1177/11786302211014401 PMC8114296 34017178

[R39] Reinl KL, Harris TD, North RL, Almela A, Berger SA, Bizic M, (2023). Blooms also like it cold. Limnol. Oceanogr. Lett. 2023:10316. doi: 10.1002/lol2.10316

[R40] Scavia D, Bertani I, Testa JM, Bever AJ, Blomquist JD, Marjorie Linker LC, (2021). Advancing estuarine ecological forecasts: seasonal hypoxia in Chesapeake Bay. Ecol. Appl. 31:eap.2384. doi: 10.1002/eap.2384 PMC8459276 34128283

[R41] Smucker NJ, Beaulieu JJ, Nietch CT, and Young JL (2021). Increasingly severe cyanobacterial blooms and deep water hypoxia coincide with warming water temperatures in reservoirs. Glob. Change Biol. 27, 2507–2519. doi: 10.1111/gcb.15618 PMC8168688 33774887

[R42] Sonak S, Patil K, and Devi (2018). Causes, human health impacts and control of harmful algal blooms: a comprehensive review. Environ. Pollut. Protect. 3, 40–55. doi: 10.22606/epp.2018.31004

[R43] National US Office for Harmful Algal Blooms (2024a). Historical Occurrence of HABs—Harmful Algal Blooms. Available at: https://hab.whoi.edu/about/historical-occurrence-of-habs (accessed March 5, 2024).

[R44] U.S. National Office for Harmful Algal Blooms (2024b). Socioeconomic—Harmful Algal Blooms. Available at: https://hab.whoi.edu/impacts/impacts-socioeconomic (accessed March 5, 2024).

[R45] United States Environmental Protection Agency (2019). Recommended Human Health Recreational Ambient Water Quality Criteria or Swimming Advisories for Microcystins and Cylindrospermopsin. EPA Report. Available at: https://www.epa.gov/sites/default/files/2019-05/documents/hh-rec-criteria-habs-document-2019.pdf (accessed January 4, 2024).

[R46] Van Dolah ER, Paolisso M, Sellner K, and Place A. (2015). Employing a socioecological systems approach to engage harmful algal bloom stakeholders. Aquat. Ecol. 50, 577–594. doi: 10.1007/s10452-015-9562-z 31588181 PMC6777728

[R47] Voogd R, de Vries JR, and Beunen R. (2021). Understanding public trust in water managers: findings from the Netherlands. J. Environ. Manag. 300:113749. doi: 10.1016/j.jenvman.2021.113749 34547569

[R48] Wells ML, Trainer VL, Smayda TJ, Karlson BSO, Trick CG, Kudela RM, (2015). Harmful algal blooms and climate change: learning from the past and present to forecast the future. Harmful Algae 49, 68–93. doi: 10.1016/j.hal.2015.07.009 27011761 PMC4800334

[R49] Wolf D, and Klaiber HA (2017). Bloom and bust: toxic algae’s impact on nearby property values. Ecol. Econ. 135, 209–221. doi: 10.1016/j.ecolecon.2016.12.007

[R50] World Health Organization (2020). Cyanobacterial Toxins: Microcystins Background Document for Development of WHO Guidelines for Drinking-Water Quality and Guidelines for Safe Recreational Water Environments. Available at: https://apps.who.int/iris/bitstream/handle/10665/338066/WHO-HEP-ECH-WSH-2020.6-eng.pdf?sequence=1&isAllowed=y (accessed August 7, 2023).

[R51] Zamor RM, Franssen NR, Porter C, Patton TM, and Hambright KD (2014). Rapid recovery of a fish assemblage following an ecosystem disruptive algal bloom. Freshw. Sci. 33, 390–401. doi: 10.1086/675508

