# Supplementary material for: Expert insights on managing harmful algal blooms
Source: Front Freshw Sci. Author manuscript; Available in PMC 2025 Oct 2. (PMC11976657; doi:10.3389/ffwsc.2024.1452344)
Supplement: Supplement1 [file NIHMS2058244-supplement-Supplement1.docx]

**Appendix 1. Full Semi-Structured Interview Questions**

*Background*

1. What is your occupation?
2. Where is your work based?
3. What are your primary concerns about water quality in local lakes and ponds?
4. What is your involvement with water quality and HABs?
5. How long have you been working on water quality related to HABs?

*Beliefs that constitute attitudes*

1. What do you see as the difference between algal blooms generally and harmful algal blooms?

We will be focused for the rest of the interview on harmful algal blooms specifically.

1. What do you know about harmful algal bloom risk in local lakes and ponds? (adapted from Deffner and Haase, 2018)
   1. If needed, how much of a concern are HABs to you for your area?
2. Have you noticed the occurrence of HABs events change in local lakes and ponds in the past 10 years? What have you noticed? If you haven’t been in this position for that long, how have you noticed HABs occurrence shift during your tenure in this position?
   1. Do you think they have increased or that our monitoring has increased?
   2. Do you think awareness of HABs has increased over the last 10 years?
3. If HABs events increased, what do you see as the negative impacts? (adapted from Jacobs and Buijs, 2011)
   1. If needed, what do you see as the negative human impacts of increased harmful algal blooms? (adapted from Jacobs and Buijs, 2011)
   2. If needed, What do you see as the negative environmental impacts of increased harmful algal blooms? (adapted from Jacobs and Buijs, 2011)
   3. What is your understanding of the risks from recreating in and on waterbodies affected by HABs?
4. What do you think is needed to handle or mitigate increased HABs events?
   1. How does consideration of human use or societal value of the water affect your recommendations?
   2. What are the major obstacles to this approach?
   3. How does this choice affect nutrient management? Hydrologic impacts?

*Public engagement*

1. What interaction – if any – do you have with members of the public? Give us an example of a positive interaction. A negative interaction.
2. Have you ever involved the public in your work? In what capacities? How has that gone? What impact have you seen that have on participants? Give me an example.
3. In general, how supportive are community members of efforts to address HABs? Are there particular communities that seem more supportive?
   1. What are constituents’ primary concerns and objections?
   2. How do you address neighboring landowners’ concerns or objections? How do you cope with conflict between the ecological objectives of the project and the values of nearby residents?
4. If the public could only understand one thing about harmful algal blooms – or the potential impacts of your work – what would you like that to be?
5. What has public engagement looked like for your work in the past?
   1. With those experiences, is there anything you would do differently in engaging the public in future efforts?
6. How do you communicate HABs events or concerns to the community?
   1. What kind of trainings/educational opportunities have you had with constituents about harmful algal blooms?
7. What do you believe are the biggest communication needs related to HABs?
8. When you think about who needs to be engaged about HABs, who are those people?

*CLOSING*

1. Is there anything you’d like to add?
2. Are there other people (managers or members of the public) that you think we should talk to for this project?
